# Influence of Aerobic Exercise After Static Stretching on Flexibility and Strength in Plantar Flexor Muscles

**DOI:** 10.3389/fphys.2020.612967

**Published:** 2020-12-03

**Authors:** Kosuke Takeuchi, Masatoshi Nakamura

**Affiliations:** ^1^Faculty of Rehabilitation, Kobe International University, Kobe, Japan; ^2^Graduate School of Comprehensive Human Sciences, University of Tsukuba, Tsukuba, Japan; ^3^Institute for Human Movement and Medical Sciences, Niigata University of Health and Welfare, Niigata, Japan

**Keywords:** stretching, aerobic execise, stiffness, passive torque, peak torque, ankle joint, electromyography

## Abstract

Aerobic exercise could improve stretch-induced strength deficits. However, mechanisms of the improvement were unclear. The purpose of the study was to examine the effects of aerobic exercise after static stretching (SS) on flexibility and isometric strength in ankle plantar-flexor muscles. Fifteen healthy males received two interventions after SS of their ankle plantar-flexor muscles for 5 min. One was aerobic exercise for 10-min on a cycling ergometer, and the other was a 10-min rest as a control. Range of motion (ROM) of ankle dorsiflexion, passive torque at terminal ROM, muscle-tendon unit (MTU) stiffness, muscle tendon junction displacement, peak torque of ankle plantarflexion, and the amplitude of electromyography (EMG) were measured. Immediately after the SS, in both interventions, ROM, passive torque, and muscle tendon junction displacement increased significantly (*p* < 0.05), while MTU stiffness, peak torque, and the amplitude of EMG were significantly decreased (*p* < 0.05). After 10-min on a cycling ergometer, the decreased peak torque and amplitude of EMG indicated higher values than those before SS (*p* < 0.05), while MTU stiffness was no change. In conclusion, SS increased ROM because of the decreased MTU stiffness as well as increased tolerance for stretching. Aerobic exercise could increase the muscle strength and amplitude of EMG which decreased after static stretching.

## Introduction

Static stretching (SS) is commonly used as part of a warm-up routine before sports activities to enhance joint range of motion (ROM; Magnusson et al., 1996; Marshall et al., 2011; Page, 2012; Young et al., 2013) and to potentially prevent sports-related injuries (Amako et al., 2003; Malliaropoulos et al., 2004; Small et al., 2008; McHugh and Cosgrave, 2010). An increase in ROM after SS is attributed to changes in passive properties of the muscle-tendon unit (MTU; Duong et al., 2001; Guissard and Duchateau, 2004; Weir et al., 2005; Morse et al., 2008) and/or tolerance for stretching (Magnusson et al., 1997; Magnusson, 1998; Nakao et al., 2019; Takeuchi and Nakamura, 2020). To evaluate the changes in the passive properties of the MTU, MTU stiffness is often calculated (Magnusson et al., 1997; Magnusson, 1998; Longo et al., 2014). MTU stiffness is calculated by using a torque-angle curve during passive joint movement, and it indicates the viscoelasticity of the MTU (Magnusson et al., 1996, 1997; Morse et al., 2008; Nakamura et al., 2011, 2013). MTU stiffness decreases effectively after more than 2 min of SS (Matsuo et al., 2013; Nakamura et al., 2019). Previous reports have shown that too much stiffness may lead to various lower body injuries including soft-tissue and joint and bone injuries, occurring in non-contact situations (Ekstrand and Gillquist, 1983; Watsford et al., 2010; Pickering Rodriguez et al., 2017). Therefore, many conditioning coaches use SS as a part of a warm-up routine in order to prevent sports-related injuries, because other interventions including aerobic exercise (Takeuchi et al., 2018), anaerobic exercise (Takeuchi et al., 2018), and dynamic stretching (Mizuno, 2017), cannot decrease the stiffness.

However, much previous research suggested that SS before sports activities should be avoided because it decreases muscle performance such as muscle strength, jump height, and muscle power (Bishop, 2003; Behm and Chaouachi, 2011; Carvalho et al., 2012; Mizuno et al., 2013a; Simic et al., 2013). A decrease in muscle performance after SS is attributed to a decrement in MTU stiffness and neural activity (Weir et al., 2005; McHugh and Cosgrave, 2010; Mizuno et al., 2013a). Therefore, a decrement in MTU stiffness after SS is effective for preventing sports-related injuries, but it causes a deficit of muscle performance.

Conventional warm-up protocol is usually comprised of low-intensity aerobic exercise, stretching, and sport-specific exercise (McCrary et al., 2015; McGowan et al., 2015). Recent reports have shown that sport-specific exercise performed after SS could restore the negative effects induced by SS on jumping (Cè et al., 2008; Taylor et al., 2009; Pearce et al., 2012), throwing (Mascarin et al., 2015), and isokinetic strength (Bengtsson et al., 2018). However, these previous studies did not examine the mechanisms of the restoration of the decrement in muscle performance. The decrement in muscle performance after SS is caused by a decrement in MTU stiffness and neural activity (Weir et al., 2005; McHugh and Cosgrave, 2010; Mizuno et al., 2013a). If the restoration of the muscle performance after the sports-specific exercise is caused by the restoration of MTU stiffness, it means that the positive effects of SS disappear after the exercises. To examine the mechanisms of restoration of the muscle performance, the effects of sports-specific exercise on MTU stiffness should be evaluated. However, it is difficult to evaluate the effects of such exercise, because the sports-specific exercise that is needed as a part of a warm-up differs between each sport (Weir et al., 2005; McHugh and Cosgrave, 2010; Mizuno et al., 2013a). On the other hand, it is shown that ROM and muscle strength increased and MTU stiffness showed no change after low-intensity aerobic exercise, which is the component of conventional warm-up protocol (Takeuchi et al., 2018). Therefore, it may be possible to clarify the mechanisms of restoration of a deficit of muscle strength induced by SS by examining the effects of aerobic exercise after SS. The purpose of the present study was to examine the effects of aerobic exercise after SS on MTU stiffness, muscle strength, and EMG activity. It was hypothesized that aerobic exercise after SS would increase muscle strength through an increase in EMG activity rather than restoration of MTU stiffness.

## Materials And Methods

### Experimental Approach to the Problem

**Figure 1:**
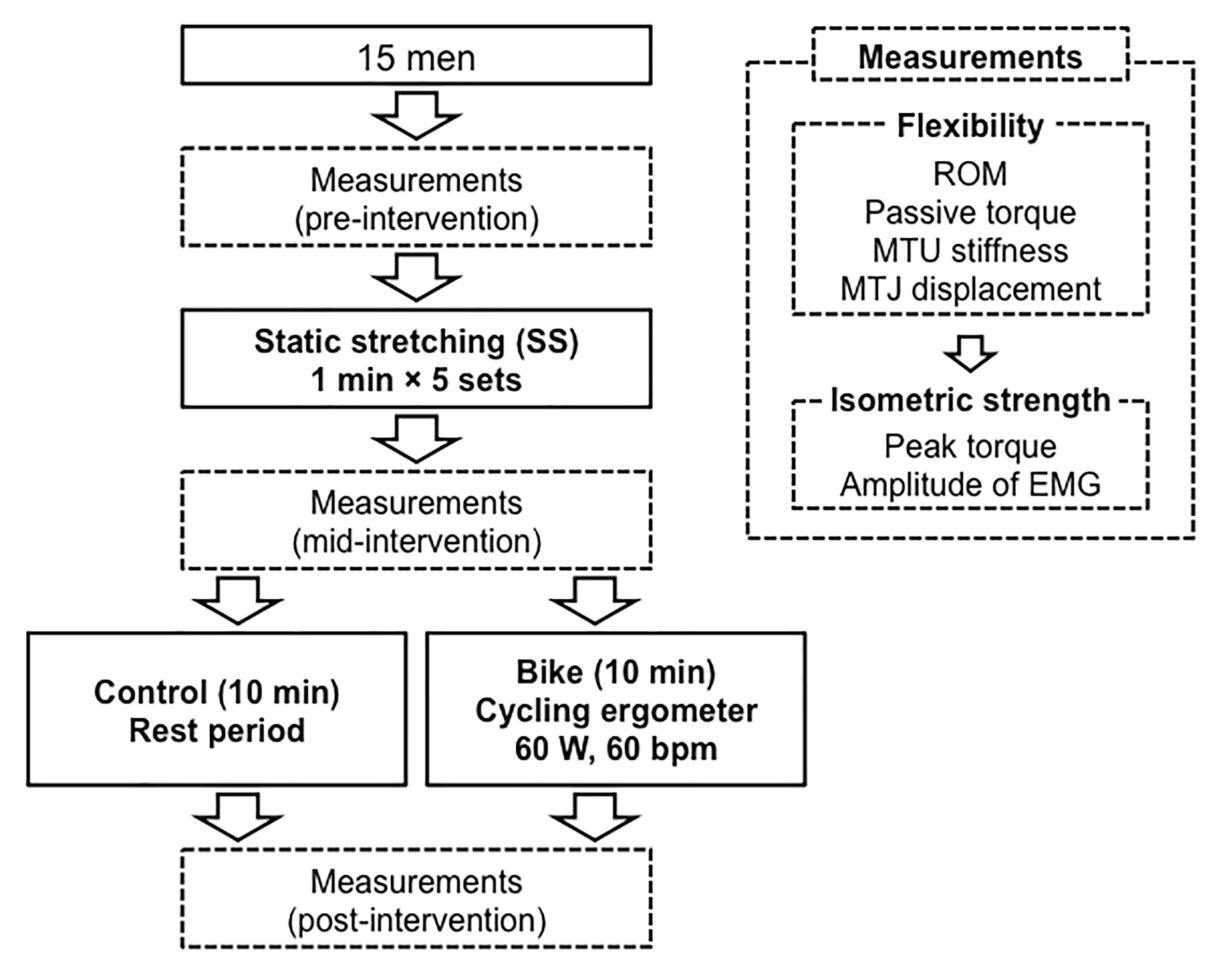
Experiment protocol. ROM, range of motion; MTU stiffness, muscle-tendon unit stiffness; MTJ displacement, muscle tendon junction displacement, EMG, electromyography.

The subjects visited the laboratory two times, with an interval of 1 week between visits, and they received the two interventions in random order: Control and Bike. One was a 10-min rest as a control, and the other was pedaling for 10-min on a cycling ergometer as aerobic exercise. Flexibility and isometric strength of the ankle joint were measured before SS, after SS, and after each intervention (pre-intervention, mid-intervention, and post-intervention, respectively). The procedure of the data collection was as follows: first measurements of flexibility and isometric muscle strength (pre-intervention), 5-min SS, second measurements (mid-intervention), a 10-min rest or 10-min on a cycling ergometer, and third measurements (post-intervention; Figure 1). This procedure was completed in the same room in which the temperature was kept at 25°C.

### Subjects

Fifteen healthy males (23.9 ± 2.2 years, 173.6 ± 5.8 cm, 68.5 ± 8.8 kg) who had not experienced any strength or flexibility training programs were recruited. Subjects with a history of lower limb pathology were excluded from the investigation. The sample size was calculated with a power of 80%, alpha error of 0.05, and effect size of 0.25 (middle) using G*Power 3.1 software (Heinrich Heine University, Düsseldorf, Germany), and the results showed that the requisite number of participants for this study was 14. All subjects were informed of the requirements and risks associated with their involvement in this study and signed a written informed consent document. The study was performed in accordance with the Declaration of Helsinki (1964). The Ethics Committee of the Biwako Seikei Sport College approved the study (No. 132).

### Measurements of Flexibility

Flexibility of the ankle joint was examined using an isokinetic dynamometer machine (Biodex System 4; Sakai Medical Co., Japan) and ultrasonography (ProSound a7; Hitachi Aloka Medical, Ltd., Japan). The subjects were secured to a calibrated dynamometer machine with the knee in full extension. The dominant foot was attached to the footplate of the dynamometer, and the ankle joint was aligned with its axis. In the present study, reported ankle dorsiflexion angles were measured using the dynamometer. A 90° angle between the footplate and floor was defined as 0° of ankle dorsiflexion/plantarflexion. The footplate passively moved from 0° to terminal ROM of ankle dorsiflexion. Terminal ROM was recorded at the maximum tolerable angle without pain. ROM of ankle dorsiflexion, passive torque of ankle plantarflexion, MTU stiffness, and muscle-tendon junction (MTJ) displacement were measured to show any alteration in flexibility. The subjects were instructed to relax during the measurements.

#### Passive Torque and MTU Stiffness

Passive torque of ankle plantarflexion was measured through the entire passive ankle dorsiflexion. In data analysis, passive torque at terminal ROM was used to examine the alteration of tolerance. Increased passive torque at terminal ROM indicated that the subjects were stretched with a higher force, which meant that an increased tolerance for stretching was obtained (Magnusson, 1998; Folpp et al., 2006; Marshall et al., 2011). The torque-angle curve during passive ankle dorsiflexion was plotted and the slope of the curve from 15 to 25° was defined as MTU stiffness (Nm/degree; Kubo et al., 2002; Nakamura et al., 2011).

#### MTJ Displacement

The B-mode of the ultrasonography was used to determine displacement of the MTJ of the gastrocnemius medialis during passive ankle dorsiflexion. The MTJ was identified according to Maganaris and Paul (1999) and visualized on a sagittal plane of the ultrasonography images using an 8 MHz linear array probe (UST-5712, Hitachi Aloka Medical. LTD, Japan). The probe was placed securely on the skin. Displacement of the MTJ was defined as the distance between a reflective marker attached to the skin and the MTJ (Kubo et al., 2002; Morse et al., 2008; Nakamura et al., 2011, 2013). Displacement of the MTJ was calculated by using software (Image J 1.45s, National Institutes of Health, United States). MTJ displacement was measured between 0° and terminal ROM at pre-intervention. In a previous study, high intraclass correlation coefficients with values of 0.985 demonstrated the reliability of the MTJ displacement measurement procedure used in this study (Nakamura et al., 2011).

### Measurement of Isometric Muscle Strength

#### Peak Torque

The measurement of isometric muscle strength was performed immediately after the measurement of flexibility. To determine isometric muscle strength, peak torque of ankle plantarflexion during maximum voluntary isometric contraction (MVIC) was measured. The subjects performed two MVIC on the dynamometer machine at 0° after the measurements of flexibility. Each MVIC was maintained for 5-s with a 2-min rest period to prevent the development of fatigue. During the MVIC, the subjects were instructed to give their maximal effort for each trial. The highest torque during MVIC was recorded, and the greatest value of the two contractions was used as isometric muscle strength.

#### Electromyography

To evaluate neural activity of the gastrocnemius muscle during MVIC, the amplitude of electromyography (EMG) was measured using bipolar, 13 mm, Ag/AgCl surface electrodes (S&ME, Tokyo, Japan) placed on the most prominent bulge of the gastrocnemius mediaris muscle. One ground electrode was positioned on the head of the fibula. At the time of measurement on the first day, the attachment site of the electrode was marked to measure the same site on the second day. The skin was abraded with sandpaper and cleaned with alcohol. The amplitude of EMG was recorded with a band width of 5~500 Hz. EMG signals were transmitted to a digital data recorder at a sampling rate of 2 kHz. The amplitude of EMG values in μV was calculated with root mean square.

### Interventions

#### Static Stretching

The repeated SS for the gastrocnemius muscle was performed on an isokinetic dynamometer machine in the same fashion as a previous study (Mizuno et al., 2013a). The ankle joint was passively moved by the dynamometer machine from 0° to the maximal ankle dorsiflexion angle. This position was held for 1 min. Thereafter, the footplate was returned to 0°. This stretching procedure was repeated five times. The maximal dorsiflexion angle was reassessed at each passive ankle dorsiflexion. The subjects were instructed to relax throughout the SS.

#### Cycling Ergometer

Aerobic exercise on a cycling ergometer was performed after the 5-min SS. The exercise consisted of 10-min of pedaling the cycling ergometer (60 W, 60 rpm; Takeuchi et al., 2018).

### Statistical Analysis

All data are reported as mean ± standard deviation. A two-way repeated ANOVA was used to examine the effects of intervention (Control vs. Bike) and time (pre-intervention vs. mid-intervention vs. and post-intervention). Post-hoc analyses were made by using Bonferroni’s test. SPSS version 21 (IBM, Tokyo, Japan) was used for all statistical analyses. Differences were considered statistically significant at a level of *p* < 0.05.

## Results

### Range of Motion

There was a significant two-way interaction (*p* = 0.04, partial eta squared = 0.22). From pre-intervention to mid-intervention, ROM significantly increased in both interventions (both, *p* = 0.01; Table 1). From mid-intervention to post-intervention, ROM decreased in Control (*p* = 0.02), but it had no change in Bike (*p* = 0.51). In both interventions, ROM at post-intervention indicated higher values compared to pre-intervention (Control, *p* = 0.02, Bike, *p* = 0.01). In post-intervention, there was no significant difference between either intervention (*p* = 0.72).

**Table 1:** Alteration in measurements of flexibility.

Values	Interventions	Pre-intervention	Mid-intervention	Post-intervention
ROM (degree)	Control	26.8 ± 7.5	31.1 ± 7.4^*^	28.9 ± 7.6^*^^,^^†^
	Bike	25.0 ± 5.9	28.9 ± 7.4^*^	29.3 ± 6.5^*^
Passive torque at terminal ROM (Nm)	Control	25.6 ± 7.3	27.1 ± 7.0^*^	27.6 ± 7.5^*^
	Bike	24.4 ± 7.4	26.5 ± 6.7^*^	28.7 ± 7.4^*^
MTU stiffness (Nm/degree)	Control	0.69 ± 0.19	0.52 ± 0.14^*^	0.54 ± 0.15^*^
	Bike	0.68 ± 0.18	0.51 ± 0.15^*^	0.53 ± 0.16^*^
MTJ displacement (cm)	Control	1.11 ± 0.29	1.31 ± 0.31^*^	1.24 ± 0.33^*^
	Bike	1.11 ± 0.32	1.22 ± 0.32^*^	1.20 ± 0.32^*^

Data were represented as mean ± standard deviation. ROM, range of motion; MTU stiffness, muscle-tendon unit stiffness; MTJ displacement, muscle-tendon junction displacement.

* *p* < 0.05 vs. pre-intervention value at the same intervention.

† *p* < 0.05 vs. mid-intervention value at the same intervention.

### Passive Torque at Terminal ROM

There was no significant two-way interaction (*p* = 0.17, partial eta squared = 0.13), and no main effect for intervention (*p* = 0.50, partial eta squared = 0.04), however, there was a significant main effect for time (*p* = 0.01, partial eta squared = 0.31; Table 1). From pre-intervention to mid-intervention, passive torque significantly increased in both interventions (*p* = 0.04). Passive torque at terminal ROM at post-intervention was higher than at pre-intervention in both interventions (*p* = 0.03).

### MTU Stiffness

There was no significant two-way interaction (*p* = 0.98, partial eta squared = 0.00), and no main effect for intervention (*p* = 0.82, partial eta squared = 0.00); however, there was a significant main effect for time (*p* = 0.01, partial eta squared = 0.46; Table 1). From pre-intervention to mid-intervention, MTU stiffness significantly decreased in both interventions (p = 0.01). From mid-intervention to post-intervention, it showed no change (*p* = 0.68). MTU stiffness at post-intervention was lower than at pre-intervention in both interventions (*p* = 0.01).

### MTJ Displacement

There was no significant two-way interaction (*p* = 0.11, partial eta squared = 0.17), and no main effect for intervention (*p* = 0.42, partial eta squared = 0.06), however, there was a significant main effect for time (*p* = 0.01, partial eta squared = 0.74; Table 1). From pre-intervention to mid-intervention, MTJ displacement significantly increased in both interventions (*p* = 0.01). MTJ displacement at post-intervention was higher than at pre-intervention in both interventions (*p* = 0.01).

### Peak Torque of Ankle Plantarflexion During MVIC

There was a significant two-way interaction (*p* = 0.03, partial eta squared = 0.25; Table 2). From pre-intervention to mid-intervention, the peak torque of ankle plantarflexion during MVIC significantly decreased in both interventions (Control, *p* = 0.04; Bike, *p* = 0.01). From mid-intervention to post-intervention, the peak torque showed no change in Control (*p* = 0.90), although it significantly increased in Bike (*p* = 0.01). The peak torque at post-intervention was lower than at pre-intervention in Control (*p* = 0.01), but it indicated higher values in Bike (*p* = 0.02). In post-intervention, there was a significant difference between both interventions (*p* = 0.03).

**Table 2:** Alteration in measurements of maximum isometric muscle strength.

Values	Interventions	Pre-intervention	Mid-intervention	Post-intervention
Peak torque (Nm)	Control	111.6 ± 32.7	93.4 ± 38.0^*^	90.6 ± 33.3^*^
	Bike	112.8 ± 33.6	93.1 ± 35.8^*^	118.5 ± 36.6^*^^,^^†^^,^^#^
Amplitude of EMG (μV)	Control	116.8 ± 44.6	85.7 ± 46.2^*^	98.7 ± 45.9^*^
	Bike	111.8 ± 45.3	83.1 ± 52.6^*^	122.9 ± 56.7^*^^,^^†^^,^^#^

Data were represented as mean ± standard deviation. EMG, electromyography.

* *p* < 0.05 vs. pre-intervention value at the same intervention.

† *p* < 0.05 vs. mid-intervention value at the same intervention.

# *p* < 0.05 vs. post-intervention value at control.

### Amplitude of EMG

There was significant two-way interaction (*p* = 0.03, partial eta squared = 0.37; Table 2). From pre-intervention to mid-intervention, the amplitude of EMG significantly decreased in both interventions (both, *p* = 0.03). From mid-intervention to post-intervention, the amplitude of EMG showed no change in Control (*p* = 0.08), although it significantly increased in Bike (*p* = 0.04). The amplitude of EMG at post-intervention was lower than at pre-intervention in Control (*p* = 0.03), although it was higher in Bike (*p* = 0.03). In post-intervention, there was a significant difference between both interventions (*p* = 0.04).

## Discussion

In the present study, ROM of ankle dorsiflexion significantly increased after the 5-min SS in both interventions. These results reflected previous studies (Kato et al., 2010, 2011; Nakamura et al., 2011; Mizuno et al., 2013a; Konrad et al., 2017; Muanjai et al., 2017). Mizuno et al. (2013b) showed that 5-min SS increased the ROM of ankle dorsiflexion. Increased ROM after SS was attributed to decreased MTU stiffness and an increased tolerance for stretching (Guissard and Duchateau, 2004; Mizuno et al., 2013a). In the present study, passive torque at terminal ROM was used to examine any alteration of tolerance for stretching (Magnusson et al., 1997; Magnusson, 1998; Nakao et al., 2019; Takeuchi and Nakamura, 2020). Increasing the passive torque indicated that the ankle joint was moved with a higher force, which meant increased tolerance for stretching was obtained. In this study, MTU stiffness decreased and passive torque increased after 5-min SS. In addition, the alterations of ROM, MTU stiffness and passive torque were maintained after both interventions. Mizuno et al. (Mizuno et al., 2013a,b) examined the time course of ROM of ankle dorsiflexion after 5-min SS. They showed that increased ROM was restored to the baseline level by 60-min after the SS, because decreased MTU stiffness and increased tolerance were restored. Based on these data, 5-min SS increased ROM through a decrease in MTU stiffness and an increase in tolerance for stretching, and the changes continued for 10 min regardless of whether aerobic exercise with a cycling ergometer was applied or not. The time course of changes in the effects of aerobic exercise after SS should be studied further.

MTJ displacement was used to examine the alteration of muscle extensibility (Morse et al., 2008; Nakamura et al., 2011, 2013; Mizuno et al., 2013a,b). Our results showed that it increased after SS. Moreover, the increment in MTJ displacement was maintained after both interventions. An increase in MTJ displacement after SS was reported in previous studies (Nakamura et al., 2011, 2013). Nakamura et al. (2013) examined the minimum time required for SS to change MTJ displacement of the gastrocnemius muscle, and they reported that SS for more than 2-min was effective. Nakamura et al. (2011) measured fascicle length and resolved fascicle length in addition to MTJ displacement and MTU stiffness after 5-min SS. They showed that MTJ displacement increased though the fascicle length and resolved fascicle length showed no change, which data indicated that the effects of SS may be associated with a change in connective tissue properties, such as the endomysium, perimysium, and epimysium, instead of lengthening muscle fiber (Nakamura et al., 2011). Our previous study showed that the MTJ displacement of the gastrocnemius muscle showed no change after aerobic exercise with a cycling ergometer for 10 min (Takeuchi et al., 2018). These data suggested that 5-min SS increased MTJ displacement and the changes continued for 10 min regardless of whether aerobic exercise was applied or not.

In the present study, the peak torque of ankle plantarflexion during MVIC decreased by approximately 17% after 5-min SS in both interventions. Mizuno et al. (2013a) examined the effects of 5-min SS on the peak torque of ankle plantarflexion during MVIC, and showed that the peak torque was decreased by approximately 11%. Previous studies reported that an altered tension-length relationship and decreased amplitude of EMG were the cause of decreased isometric strength after SS (Magnusson et al., 1996; McHugh and Cosgrave, 2010). Decreased MTU stiffness indicated an altered tension-length relationship. In the present study, MTU stiffness and amplitude of EMG were decreased after 5-min of SS in both interventions. The results indicated that the decrease of the peak torque during MVIC was attributed to both a decrease in MTU stiffness and the amplitude of EMG. Aerobic exercise for 10 min on a cycling ergometer increased the peak torque and decreased after the SS, but it showed no change after the rest period. After the aerobic exercise on the cycling ergometer, MTU stiffness showed no change, though the amplitude of EMG was increased. These data showed that aerobic exercise with a cycling ergometer improved the stretch-induced strength deficit because of an increase in the amplitude of EMG.

A few limitations of the present study need to be considered. Firstly, this study used a cycling ergometer as an aerobic exercise because our previous study showed that it increased isometric strength in ankle plantar-flexor muscles (Takeuchi et al., 2018). However, most sport warm-ups include gross and generalized whole-body warm-ups such as dynamic mobility and skill-specific progressions. A specific warm-up may provide ergogenic benefits in addition to those provided by a general active warm-up (Bishop, 2003). Therefore, further research is required to establish the effects of gross skill-specific warm-ups. Secondly, the present study measured the amplitude of EMG of only the gastrocnemius muscle though there are many ankle plantar flexors such as soleus muscles. Thus, it was unclear whether there are differences in the amplitude of EMG between each plantar flexor muscles.

In conclusion, 5-min SS increased ROM through a decrease in MTU stiffness and an increase in tolerance. The peak torque of ankle plantarflexion during MVIC decreased after 5-min SS because of a decrease in MTU stiffness and amplitude of EMG. Aerobic exercise for 10-min on a cycling ergometer increased the decreased peak torque through an increase in the amplitude of EMG. These data suggested that when coaches use SS as part a warm-up routine, they should use aerobic exercise after SS.

## Data Availability Statement

The raw data supporting the conclusions of this article will be made available by the authors, without undue reservation.

## Ethics Statement

The studies involving human participants were reviewed and approved by Biwako Seikei Sport College. The patients/participants provided their written informed consent to participate in this study.

## Author Contributions

All authors listed have made a substantial, direct and intellectual contribution to the work, and approved it for publication.

### Conflict of Interest

The authors declare that the research was conducted in the absence of any commercial or financial relationships that could be construed as a potential conflict of interest.
